# Metabolomic Signature in Sera of Multiple Sclerosis Patients during Pregnancy

**DOI:** 10.3390/ijms19113589

**Published:** 2018-11-14

**Authors:** Claudia Rossi, Ilaria Cicalini, Mirco Zucchelli, Maria di Ioia, Marco Onofrj, Luca Federici, Piero Del Boccio, Damiana Pieragostino

**Affiliations:** 1 Department of Medical, Oral and Biotechnological Sciences, “G. d’Annunzio” University of Chieti-Pescara, 66100 Chieti, Italy; claudia.rossi@unich.it (C.R.); ilaria.cicalini@unich.it (I.C.); m.zucchelli@unich.it (M.Z.); luca.federici@unich.it (L.F.); 2 Analytical Biochemistry and Proteomics Laboratory, Research Centre on Aging and Translational Medicine (Ce.S.I-MeT), University “G. d’Annunzio” of Chieti-Pescara, 66100 Chieti, Italy; piero.delboccio@unich.it; 3 Department of Neurosciences and Imaging, University “G. d’Annunzio” of Chieti-Pescara, 66100 Chieti, Italy; maria.diioia@unich.it (M.d.I.); marco.onofrj@unich.it (M.O.); 4 Department of Pharmacy, University ‘‘G. d’Annunzio’’ of Chieti-Pescara, 66100 Chieti, Italy

**Keywords:** metabolomics, multiple sclerosis, mass spectrometry, pregnancy, ceramides, amino acids, acylcarnitines, steroids, estrogens, biomarkers

## Abstract

Multiple sclerosis (MuS) is an autoimmune disease of the central nervous system characterized by neuroinflammation, neurodegeneration, and degradation of the myelin sheath. Epidemiological studies have shown that the female gender is more susceptible than the male gender to MuS development, with a female-to-male ratio of 2:1. Despite this high onset, women have a better prognosis than men, and the frequency of the relapsing phase decreases during pregnancy, while it increases soon after birth. Therefore, it is interesting to investigate hormonal fluctuations during pregnancy and whether they correlate with metabolic signatures. To gain a deeper inside into the biochemical mechanism of such a multifactorial disease, we adopted targeted metabolomics approaches for the determination of many serum metabolites in 12 pregnant women affected by MuS by mass spectrometry analysis. Our data show a characteristic hormonal fluctuation for estrogens and progesterone, as expected. They also highlight other interesting hormonal alterations for cortisol, corticosterone, 11-deoxycortisol, 4-androstene-3,17-dione, testosterone, and 17α-hydroxyprogesterone. Furthermore, a negative correlation with progesterone levels was observed for amino acids and for acylcarnitines, while an imbalance of different sphingolipids pathways was found during pregnancy. In conclusion, these data are in agreement with the characteristic clinical signs of MuS patients during pregnancy and, if confirmed, they may add an important tessera in the complex mosaic of maternal neuroprotection.

## 1. Introduction

Multiple sclerosis (MuS) is an autoimmune disease of the central nervous system (CNS) characterized by neuroinflammation, neurodegeneration, and loss of the myelin sheath of axons [1]. Genetic susceptibility, environmental factors, and their interactions are involved in the pathogenesis of MuS. On the other hand, clinical experience suggests the study of sex differences as a source of important insight into the mechanisms underlying the pathogenesis and progression of this chronic inflammatory disease of the CNS [2]. Epidemiological studies demonstrated that MuS is more frequent in women, with an incidence two times higher than in men [3]. Moreover, the age of MuS onset is variable, but the disease, predominantly, occurs in women [2] during childbearing years, between 20 and 40 years of age. Thus, the effect of pregnancy on MuS is an important aspect to be explored [4]. It should also be emphasized that the prognosis for MuS is more favorable in women than in men and that pregnancy strongly affects disease activity, leading to a decrease of the frequency of relapsing phases during the third trimester of pregnancy, which increases again soon after birth [5], showing a direct proportionality to progesterone and estrogen fluctuations [6]. As also reported by Voskuhl R and Momtazee C [4], it is worth noting that MuS pregnant patients showed an approximate 70% reduction in relapse rates during the third trimester of pregnancy. The hormonal fluctuations described during pregnancy and, in particular, the increase in estrogens and progesterone, with a peak in the last trimester, have been shown to be neuroprotective in several neurological disease models [7,8]. Indeed, it should be mentioned that the protective effect during the third trimester of pregnancy and the marked worsening of symptoms post-partum and also during menopause have spurred several research teams to study alternative sex steroids therapies for MuS [9]. Considering the many changes occurring in pregnancy, it is intriguing to investigate whether these hormonal fluctuations could correlate with other metabolic alterations involved in specific biochemical mechanisms. In order to gain further insights into MuS during pregnancy, we started collecting and analyzing sera of MuS women in each trimester of pregnancy (First trimester: T1, Second trimester: T2, Third trimester: T3) and post-partum for studying metabolites levels. As it has been already discussed, estrogens and progesterone increase during pregnancy, particularly in the third trimester, the time of greatest disease protection. Thus, in this study, we firstly propose targeted metabolomics approaches for the determination of estrogens such as estradiol (E2) and estrone (E1) and of seven steroids, including cortisol (CORT), corticosterone (CCONE), 11-deoxycortisol (11-DECOL), 4-androstene-3,17-dione (ADIONE), testosterone (TESTO), 17α-hydroxyprogesterone (17-OHP), and progesterone (PROG). Additionally, steroids quantification was integrated with that of other MuS-related metabolites, such as sphingolipids and ceramides,14 amino acids (AAs), free carnitine (C0), 35 acylcarnitines (ACCs), succinyl acetone (SA), 2 nucleosides, and 4 lysophospholipids (LPC).

## 2. Results

### 2.1. Serum Metabolomics Investigation during Pregnancy in Multiple Sclerosis

**Figure 1:**
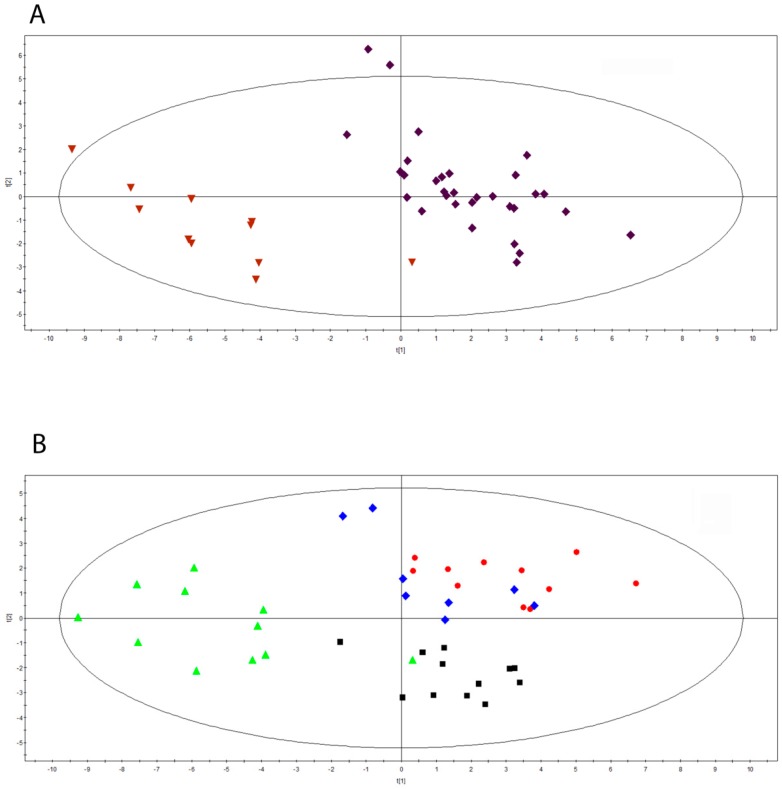
(**A**) Scores scatter plot calculated on two components by using 42 observation of 12 MuS cases, divided into two time groups: during pregnancy and post-partum. The classification is based on each metabolite concentration, excluding steroids and estrogens. The Partial Least Squares Discriminant Analysis (PLS-DA) was used to classify sera from MuS patients in each time group as follow: purple diamonds: during pregnancy; dark red inverted triangles: post-partum obtained by R2Y = 0.865 and Q2(cum) = 0.758. (**B**) Scores scatter plot calculated on two components by using 42 observations divided into three different times of pregnancy and the post-partum period. classification is based on each metabolite concentration, excluding steroids and estrogens. The Partial PLS-DA was used to classify sera from MuS patients in each time group as follow: black boxes: first trimester of pregnancy; red dots: second trimester of pregnancy; blue diamonds: third trimester of pregnancy; green triangles: post-partum obtained by R2Y = 0.484 and Q2(cum) = 0.321.

We performed targeted metabolomics approaches for the determination of many serum metabolites such as steroids, estrogens, sphingolipids, ceramides, AAs, ACCs, SA, nucleosides, and LPCs, as reported in the Materials and Methods Section. Partial Least Squares Discriminant Analysis (PLS-DA) was performed to classify the pregnancy period and the post-partum one, after the exclusion of steroids and estrogens. As shown in Figure 1A, the Scores scatter plot calculated on two components by using 42 observations of 12 MuS cases, divided into two time groups, highlights an evident segregation between the groups considered (R2Y = 0.865 and Q2(cum) = 0.758). Surprisingly, these data show a strong lipid and amino acids modulation in pregnancy of MuS patients. Figure 1B depicts the PLS-DA obtained by dividing the 42 observations into four groups, namely, the three trimesters of pregnancy and the post-partum period. The multivariate analysis showed an unambiguous separation between the four clinical groups analyzed (black boxes: first trimester of pregnancy; red dots: second trimester of pregnancy; blue diamonds: third trimester of pregnancy; green triangles: post-partum), already highlighting a trend of modulation during pregnancy. Moreover, the same trend observed in Figure 1B was found by including in such analysis estrogens and steroids (Figure S1). The most important metabolic variables in this segregation are listed in Table 1. Intriguingly, the most significant metabolites, as listed in Table 1, are sphingolipids and ceramides.

### 2.2. Characterization of Hormonal Fluctuations during Pregnancy in Multiple Sclerosis

**Figure 2:**
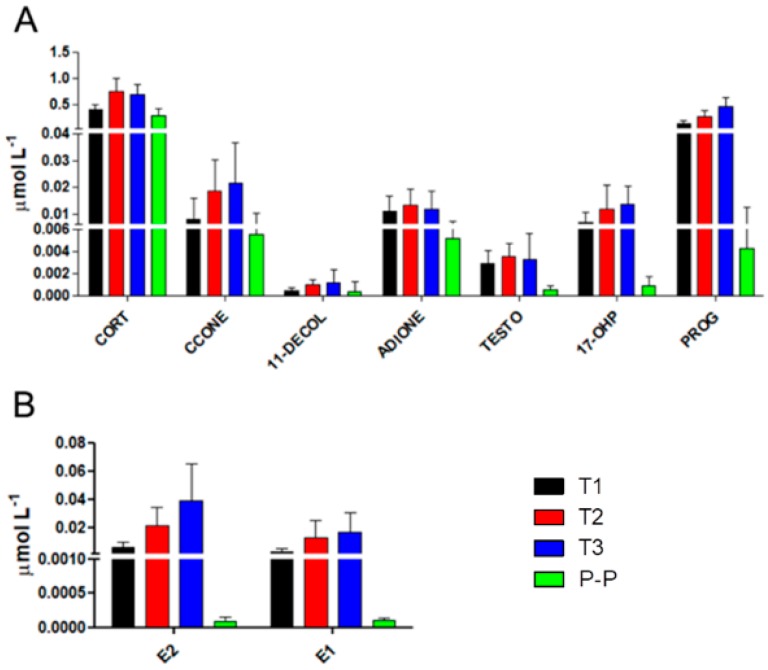
(**A**,**B**) Concentrations of steroids and estrogens in each of the three trimesters of pregnancy and post-partum (P-P) as follow: black bars for the first trimester of pregnancy, red bars for the second trimester of pregnancy, blue bars for the third trimester of pregnancy, and green bars for the post-partum period. Data are mean values, and bars represent the corresponding standard deviations (SD). The significance, expressed in terms of *p*-value, is reported in Table 2 for each time comparison for the observed steroids and estrogens. CORT: cortisol, CCONE: corticosterone, 11DECOL: 11-deoxycortisol, ADIONE: 17-dione, TESTO: testosterone, 17-OHIP: 17α-hydroxyprogesterone, PROG: progesterone, E2: estradiol, E1: estrone.

**Figure 3:**
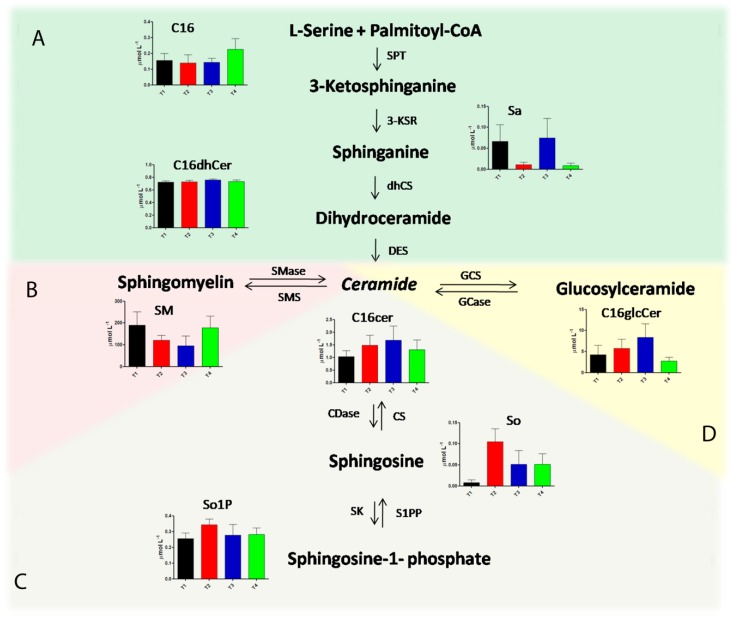
Sphingolipid metabolic pathways and concentration levels of the related metabolites in each of the three trimesters of pregnancy and post partum as follow: black bars for the first trimester of pregnancy, red bars for the second trimester of pregnancy, blue bars for the third trimester of pregnancy, and green bars for the post-partum period. The de novo synthesis pathway is reported in Panel (**A**), the sphingomyelin pathway is represented in Panel (**B**), the catabolic pathway is schematized in Panel (**C**), and glucosylceramide synthesis is summarized in Panel (**D**). SPT: serinepalmitoyltransferase; 3-KSR: 3-ketosphinganine reductase; CS: ceramidesynthetase; DES: dihydroceramidedesaturase; Smase: sphingomyelinase; SMS: sphingomyelinsynthetase; Cdase: ceramidase; CS: ceramide synthase; SK: spingosine kinase; S1PP: spingosine-1-phosphate phosphatase; GCS: glycosylceramide synthase; Gcase: glycosylceramidase.

After LC-MS/MS analysis for the quantification of steroids and estrogens in serum samples of MuS patients during pregnancy and post-partum, as expected, several significant differences were observed. Figure 2 Panels A and B show the hormonal fluctuations of pregnancy, in order to define the distribution of data in each trimester of pregnancy and in the post-partum period: with the exception for CORT and ADIONE in T3, a gradual increase in each trimester of pregnancy was observed for all the steroids and estrogens we monitored, while an important decrease was revealed in the post-partum period, as highlighted by the significance of the post hoc test between T3 and post-partum. The observed differences, in terms of *p*-value, in each time comparison for steroids and estrogens are reported in Table 2. The most significant decreases in the post-partum period were obviously observed for 17-OHP, PROG, E2, E1, and, surprisingly, also for CORT, as highlighted in Figure 3 and in Table 2.

### 2.3. Alteration in Sphingolipids and Ceramides Metabolic Pathways during Pregnancy in Multiple Sclerosis

Following the quantification of different serum sphingolipids and ceramides by LC-MS/MS analysis, many interesting alterations in sphingolipid metabolism were identified during the trimesters of pregnancy and in the post-partum period. Figure 3 represents the metabolic pathways involved in sphingolipid metabolism, in particular, the de novo synthesis pathway, sphingomyelin pathway, catabolic pathway, and glucosylceramide synthesis pathway, as described in Panels A–D, respectively. For each quantified metabolite, a histogram showing the concentration levels in the monitored times of pregnancy is reported. Specifically, C16, Sa, and C16dhCer trends are shown in Panel A, highlighting a significant accumulation of Sa in the third trimester of pregnancy (T3), with a strong decrease in the post-partum period. SMs levels were significantly decreased during pregnancy, especially in T3, as reported in Panel B. Concerning the catalytic pathway (Panel C), an accumulation of So and So1P in T2 and, subsequently, a significant decrease of these metabolites in T3, also maintained in the post-partum period, were observed. Finally, we found a gradual increase of C16glcCer during the first, second, and third trimesters of pregnancy, followed by a dramatic decrease of this metabolite in the post-partum period, as depicted in Panel D. In particular, C16Cer levels, which is located at the junction of the metabolic pathways involved in sphingolipid metabolism, significantly increased in the second and in the third trimesters of pregnancy, compared with the first trimester. Moreover, in the post-partum period, we found a significant correlation with the Expanded Disability Status Scale (EDSS) for C16Cer and C18Cer (Figure S2).

### 2.4. Serum Amino Acids and Acylcarnitines Profiles of Multiple Sclerosis in Pregnancy

**Figure 4:**
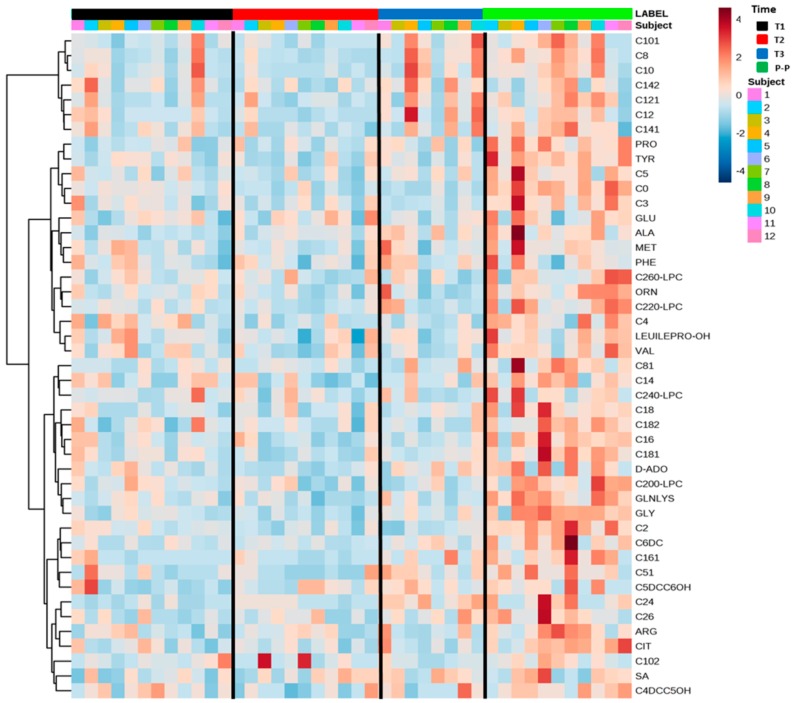
The heatmap displays data related to amino acids (AAs), free carnitine (C0), acylcarnitines (ACCs), succinyl acetone (SA), nucleosides, and lysophospholipids (LPCs) in the form of colored cells. It provides direct visualization of the relative levels of individual samples and time series. At the top of the image, the black bar indicates the first trimester of pregnancy (T1), the red bar indicates the second trimester of pregnancy (T2), the blue bar indicates the third trimester of pregnancy (T3), and the green bar indicates the post-partum period. The color code describes high serum levels (red) and low serum levels (blue) of each metabolite.

The heatmap visualization (Figure 4), a commonly used feature for clustering, showed a general and constant increase in the serum levels of AAs, C0, ACCs, SA, nucleosides, and LPC in the post-partum period of MuS patients. The red cells present in the last column related to the post-partum period highlight the described trend. In particular, by looking at the most important metabolic variables for these classes of metabolites (Table 1), it is possible to observe a strong significant difference between the levels of C0, C2, C4, Gly, Tyr, Pro in the second-third trimester (T2/T3) of pregnancy and those the post-partum period. Ala gradually increased from T2 to post partum, showing a significant difference at these times points in the comparison with the first trimester of pregnancy (T1). Moreover, some other metabolites (such as Glu, Phe, Leu/Ile/Pro-OH, Met, most medium-chain ACCs, SA, LPCs, and nucleosides) quantified by direct infusion mass spectrometry (DIMS) analysis, showed little or no significance in the same comparison, even if their levels were increased in the post-partum period. Interestingly, in the post-partum period, we found a significant correlation with the EDSS for C16, C24, and C26 (Figure S2).

### 2.5. A Specific Metabolic Profile Correlates with Progesterone Levels

**Figure 5:**
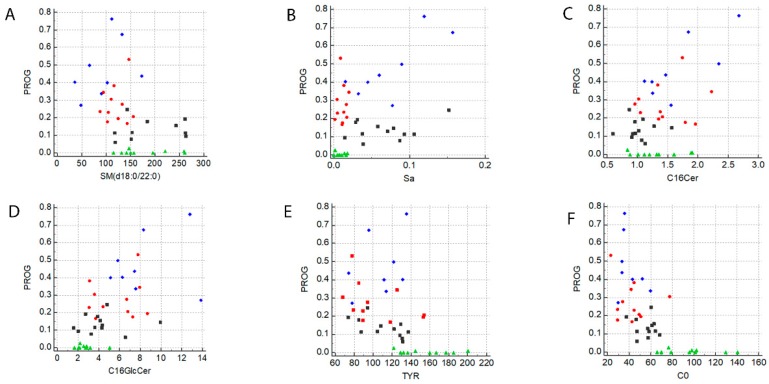
Panels (**A**–**F**) show the Rank correlations, performed by using MedCalc 7.6, between Progesterone (PROG), Sphingomyelin (d18:0/22:0) (SM), Sphinganine (Sa), Ceramide (d18:1/16:0) (C16Cer), Glucosylceramide (d18:1/16:0) (C16GlcCer), Tyrosine (TYR) and Free Carnitine (C0), respectively. The black squares indicate the first trimester of pregnancy, the red circles indicate the second trimester of pregnancy, the blue diamonds indicate the third trimester of pregnancy, and the green triangles indicate the post-partum period.

In order to highlight a possible correlation between the metabolites investigated in the present study and PROG levels during pregnancy in MuS patients, a Spearman non-parametric correlation test was performed. As reported in Table S1, many metabolites showed a significant positive or negative correlation with PROG. In details, a negative correlation was found for SMs, AAs, C0, ACCs, and LPCs, while a positive correlation was observed for Sa and ceramides. We listed in Table S1 the significant metabolite correlations obtained (in bold character) and, among them, we chose to represent the most interesting ones (indicated as * in Table S1) in Figure 5 Panels A–F. Each metabolite reported in Figure 5 shows a strong correlation with PROG levels during the first (T1), second (T2), and third (T3) trimester of pregnancy, while the post-partum period seems not to be influenced by PROG fluctuations.

## 3. Discussion

In this study, 12 women affected by MuS were monitored during the three trimester of pregnancy and in the post-partum period through different targeted metabolomics approaches by mass spectrometry analysis of their serum samples, in order to highlight a typical metabolic signature. Our data show a characteristic hormonal fluctuation, as expected, for estrogens (E1, E2) and PROG, while describing other interesting hormonal alterations for CORT, CCONE, 11-DECOL, 17-OHP, and TESTO. Since, the increase of estrogens and PROG have been already correlated with maternal immunomodulation, our data show for the first time that also other hormones may be involved in such immunomodulated neuroprotection [4]. In fact, the treatments with estrogens currently being used [8] have already demonstrated a possible mechanism of neuroprotection in MuS. Looking at our data on hormonal alterations in pregnancy, it may be hypothesized that also other steroids of that pathway may have a crucial role in neuroprotection and might be considered as potential pharmacological targets. Moreover, in order to gain further insights in the neuroprotection mechanisms during pregnancy and in the metabolic alterations characterizing the precipitous relapse in the post-partum period of MuS patients, we also monitored differences in other metabolic classes, such as sphingolipids, ceramides, AAs, ACCs, SA, nucleosides, and LPCs. Importantly, our results show that also these metabolites are able to segregate pregnancy from the non-pregnant condition, as well as to stratify the three trimesters of pregnancy, suggesting a complex fluctuation of the entire metabolic pattern. The segregation we described seems to be strongly influenced by an increased amino acid catabolism during pregnancy, followed by a significant increase in the levels of AAs in the post-partum period, as shown by our results. It has been already reported that the increase of amino acid catabolism may support immune homeostasis, preventing autoimmunity [10]. Indeed, a negative correlation with PROG levels was observed for AAs and also for ACCs. Actually, ACCs gradually decreased during pregnancy, as already known [11], while their increase in the post-partum period can be linked to oxidative stress [12] and to the resulting inflammation. We may thus speculate that such decrease in ACCs levels could prevent the activation of proinflammatory signaling pathways [13]. Surprisingly, sphingolipids show the most significant results in the classification of pregnancy and post-partum period in MuS patients. The role of sphingolipids in MuS is well known [14], and, in the present study, we also observed an unbalance of sphingolipid metabolism with a specific alteration at the time of pregnancy. We have previously described an enhanced release of acid sphingomyelinase-enriched exosomes, which generated a lipidomic signature in the cerebrospinal fluid of MuS patients [15]. Here, we found an accumulation of C16GlcCer in the third trimester of pregnancy and a decrease of SM, So, and So1P, probably suggesting an activation of the glucosylceramide synthesis pathway. The last trimester of pregnancy is roughly equal to the most effective disease-modifying treatments for MuS, with a low relapse rate [4], thus suggesting a potential role for GlcCer in managing such a disease. Actually, GlcCer activates iNKT cells in a CD1d-dependent manner [16]. NKT lymphocytes have been implicated in the regulation of the autoimmune process in both mice and humans, also leading to a rapid amplification of IFNgamma and IL4 after their activation [17]. It is worth noting that extracellular vesicles containing IL4 modulate neuroinflammation and significantly reduce the clinical signs in a mouse model of MuS [18]. Moreover, it was demonstrated that oral administration of GlcCer inhibits inflammation and leukocyte infiltration in a murine model [17]. In addition, as reported by Lawrence G. Miller Jr. et al., the elevation of So levels in an animal model of Experimental autoimmune encephalomyelitis (EAE) spinal cord, caused by de novo synthesis of ceramide and modulated by serine-palmitoyltransferase (SPT) activation, evolved to oligodendrocyte cell death that contributes to progressive demyelination in MuS [19]. According to these data, we found a drastic increase of Sa in the third trimester of pregnancy, in agreement with a possible block of ceramides de novo synthesis, probably linked to a mechanism of neuroprotection. In order to confirm these data, we should follow a larger cohort of patients during pregnancy. In fact, a positive correlation with PROG levels was observed for Sa and ceramides. Intriguingly, a significant correlation with EDSS was found for C16Cer, C18Cer, C16, C24, and C26 (Figure S2), providing a link between the metabolic profile and the clinical outcomes registered in the post-partum period. We would like to specify that the collection of four samples for each MuS patient during that particular time was difficult because of uneasy compliance, resulting in the loss of time points.

In conclusion, bearing in mind the limitations of the present study due to the small cohort analyzed and the lack of time points for some MuS patients, our data are in agreement with the characteristic clinical condition of MuS patients during pregnancy and they add an important element for the comprehension of the mechanism underlying maternal neuroprotective immunomodulation.

## 4. Materials and Methods

### 4.1. Ethics Statement

The study design was made following the guidelines of the local Ethics Committee that approved the study (n. 18 of 31 October 2013, protocol n. 176, Ethic committee of “G. d’Annunzio” University and ASL N.2 Lanciano-Vasto-Chieti, Italy) and conducted according to the Declaration of Helsinki (World Medical Association, 1997). All patients were informed about the procedures and provided written informed consent to participate in the study. In order to protect human subject identity, a number code was employed for specimen identification.

### 4.2. Patients

MuS patients, diagnosed according to the 2010 Polman’s criteria [20], were included in this study. Clinical diagnosis was confirmed by MRI studies and by the presence of oligoclonal bands in the cerebrospinal fluid (CSF). The EDSS score was obtained at the time of lumbar puncture. For this study, 12 MuS women were monitored in each trimester of pregnancy and for three months after giving birth. In particular, we collected 12 serum samples from MuS women in T1, 11 serum samples from MuS women in T2, 8 from MuS women in T3, and 11 from MuS women in the post-partum period. None of the patients were under therapy during pregnancy. The patients were Caucasian subjects, all with relapsing–remitting MuS, aged between 26 and 37 years, and 83% of the subjects were at their first pregnancy. No subject showed thyroid alterations and obesity signs. Details are reported in Table S2.

### 4.3. Samples Collection

Sera from MuS patients were collected at the Multiple Sclerosis Center of Chieti (Italy). All samples were maintained at 23 ± 1 °C allowing coagulation and were centrifuged at 4 °C for 15 min at 1400× *g*. Sera were recovered, collected in polypropylene tubes, and snap-frozen at −80 °C.

### 4.4. Materials

Cortisol (CORT), corticosterone (CCONE), 11-deoxycortisol (11-DECOL), 4-androstene-3,17-dione (ADIONE), testosterone (TESTO), 17α-hydroxyprogesterone (17-OHP), progesterone (PROG), estrone (E1), and estradiol (E2) were purchased from Sigma-Aldrich^®^ (St. Louis, MO, USA). ^2^H_3_-CORT, ^2^H_8_-CCONE, ^2^H_5_-11-DECOL, ^2^H_5_-ADIONE, ^2^H_5_-TESTO, ^2^H_8_-17-OHP, ^2^H_9_-PROG were from CHS^TM^ MSMS Steroids Kit, PerkinElmer^®^ (Turku, Finland). ^2^H_4_-E1, ^2^H_4_-E2 were from Cambridge Isotope Laboratories, Inc. (Andover, MA, USA). Sphinganine d18:0 (Sa), Sphinganine-1-phosphate (Sa1P), sphingosine d18:1 (So), sphingosine-1-phosphate (So1P), Ceramide (d18:1/16:0) (C16Cer), Ceramide (d18:1/18:0) (C18Cer), Ceramide (d18:1/22:0) (C22Cer), Ceramide (d18:1/24:0) (C24Cer), Ceramide (d18:1/16:0) (C16Cer), dihydroceramide (d18:1/16:1) (C16dHCer), dihydroceramide (d18:1/24:1), glucosylceramide (d18:1/16:0) (C16GlcCer), ISs Sphinganine d17:0 (d17Sa), Sphinganine-1-phosphate d17:0 (d17Sa1P), sphingosine d 17:1 (d17So), sphingosine-1-phosphate d17:1 (d17So1P), Ceramide (d18:1/17:0) (C17Cer), and glucosylceramide (d18:1/17:0) (C17GlcCer) were purchased from Avanti Polar Lipids. Water, methanol (MeOH), acetonitrile (ACN) LC-MS grade were from Romil-Pure chemistry^®^ (Cambridge, UK). Formic acid, ethanol LC-MS grade, methyl tert-butyl ether, dansyl chloride, ammonia, and dimethyl sulfoxidewere from Sigma-Aldrich^®^ (St. Louis, MO, USA). HPLC solvent additive was from CHS MSMS Steroids Tool Box, PerkinElmer^®^ (Turku, Finland). The internal standards, the extraction solution, and the quality controls (QCs) related to amino acids (AAs), free carnitine (C0), acylcarnitines (ACCs), ketones as succinyl acetone (SA), nucleosides, and phospholipids were obtained from the NeoBase 2 Non-derivatized MSMS Kit (Perkin Elmer Life and Analytical Sciences, Turku, Finland).

### 4.5. Standard Solutions, Calibrators, and Quality Controls

Each endogenous steroid stock solution (1 mg) was stored at −20 °C, after being dissolved in ethanol. Stock solutions of all compounds were diluted in methanol:water 50:50, reaching a final concentration of 0.03 µM. The internal standard mixture (IS mix) from PerkinElmer^®^ kit was prepared in 1.25 mL of ACN. Each solution was stored at −20 °C. The Daily Precipitation Solution (DPS) containing Internal Standards was prepared by diluting 1:100 the IS mix with ACN, with 0.1% formic acid. Calibrators and QCs were in human serum and were from CHSTM MSMS Steroids Kit, PerkinElmer^®^ (Turku, Finland). Concentration levels (µM) for calibrators and QC materials of each steroid monitored in the LC-MS/MS method of analysis are listed in Supplementary Table S3. Moreover, stock solutions, 1 mg/mL in ethanol, were prepared for E1, E2, 2H4-E1, and 2H4-E2 and stored at −20 °C until required. The stock solutions of each compound were diluted in an equal volume of ammonia (0.1%) and dansyl chloride in acetonitrile (0.5 mg/mL) at a final concentration of 0.02 µM (tuning solution). Working solutions for estrogens were prepared by diluting the stock solutions in dimethyl sulfoxide to achieve a final concentration of 0.02 µM. Subsequent dilutions of the working solutions in dimethyl sulfoxide were carried out to achieve solutions of estrogens with final concentrations of 5, 2, 1, 0.5, 0.2, 0.1, 0.05, 0.02, 0.01, and 0.005 µM. Calibrators in water were prepared from the above-described solutions of estrogens in dimethyl sulfoxide to achieve the concentrations of 0.05, 0.1, 0.2, 0.5, 1.0, 2.0, 5.0, 20.0, 100 nM. Similarly, QCs of estrogens in water were prepared at the following concentrations: 0.15, 1.5, and 37 nM. Regarding sphingolipid and ceramide analyses, solutions at 2.5 mg/mL in chloroform:methanol 2:1 *v/v* (stock solutions) were prepared and stored at −20 °C until required. Seven working solutions were prepared by diluting the stock solutions in methanol. Each working solution was diluted 100-fold to achieve the calibrator and QC final concentrations reported in Supplementary Table S4.

### 4.6. Sample Preparation and LC-MS/MS Analysis for Steroids Determination

For steroids quantification by LC-MS/MS analysis, 200 µL of DPS containing IS was added into each 2.0 mL microcentrifuge tube (Eppendorf^®^, Hamburg, Germany). Subsequently, 100 µL of serum sample from each patient, calibrators, and QCs was added into a tube with DPS, mixed (20 °C, 15 min) in a Thermomixer (Eppendorf^®^), and then centrifuged (4210 rcf, 20 °C, 30 min). The organic layer (175 µL) was recovered, dried in a SpeedVac, and reconstituted with 125 µL of H_2_O:MeOH 60:40. The obtained solution was gently mixed in a Thermomixer (20 °C, 15 min) and briefly centrifuged, and the supernatant was transferred into a polypropylene vial (Waters Corporation, Milford, MA, USA). The vials were placed in the autosampler for LC-MS/MS analysis. The LC-MS/MS system consisted of a HPLC Alliance HT 2795 Separations Module coupled to a Quattro UltimaPtESI tandem quadrupole mass spectrometer (Waters Corporation, Milford, MA, USA), operating in positive electrospray ionization mode with MassLynx v4.1 software (Waters). An amount of 50 µL was injected, with a total run time of 18.00 min. A detailed description of the LC-MS/MS analysis for the determination of steroids and the instruments parameters have already been reported [21]. QuanLynx 4.1 software (Waters Corporation, Milford, MA, USA) was used for data processing and quantification.

### 4.7. Sample Preparation and LC-MS/MS Analysis for Estrogens Determination

For estrogens determination by LC-MS/MS analysis, 200 µL from each serum sample, calibrators, and QCs, after adding 20 µL of internal standard mix (2H4-E1 and 2H4-E2) at 500 pg/mL, was treated with methyl tert-butyl ether (1 mL), vortex-mixed for 30 s, and centrifuged at 16,400 rpm for 20 min at 4 °C. The supernatant (600 µL) was removed, dried, and reconstituted in 50 µL of ammonia (0.1%) plus 0.5 mg/mL dansyl chloride in acetonitrile (50 µL). The samples were then incubated at 60 °C for 10 min, gently mixed, and allowed to cool to room temperature prior to analysis. The same LC-MS/MS system as for steroids determination was used for estrogens analysis. For HPLC analysis, the Atlantis dC18 3 µm C8, 2.1 × 100 mm column (Waters) with C18 4 × 2.0 mm as precolumn (Phenomenex^®^, Torrance, CA, USA) was used. An amount of 40 µL as injected and separated using the following mobile phases: water (solvent A) and acetonitrile (solvent B), both with 0.1% formic acid. The initial solvent composition was maintained at 95% A and 5% B for 9 min. The mobile phase gradient profile involved the following steps: increasing to 70% B after 9 min; then to 100% B at 15 min, holding for 5 min before returning to the initial gradient. The total run time was 25 min. The flow rate was 0.2 mL/min at 30 °C. The mass spectrometer ionization source settings were optimized as previously reported [22]. The parameters for the multiple reaction monitoring (MRM) experiments created for each analyte are summarized in Supplementary Table S5. Data processing and quantification were performed through QuanLynx 4.1 software. Linear regression with reciprocal fit weighting was used for calibration to ensure maximum accuracy at the lower concentration range [23].

### 4.8. Sample Preparation and LC-MS/MS Analysis for Sphingolipids and Ceramides Determination

For sphingolipids and ceramides determination, the LC-MS/MS analysis was performed following the extraction procedure of Si Mi et al. [24]. Briefly, 50 µL from each serum sample, calibrators, and QCs was mixed to 300 µL of chloroform:methanol 2:1 *v/v* with the internal standards mix at 0.1 µg/mL for d17Sa and d17So, at 0.2 µg/mL for d17Sa1P and d17So1P, and at 1.0 µg/mL for C17GlcCer and C17Cer. The solution was vortex-mixed for 30 s and centrifuged at 16,000× *g* rcf for 15 min at 4 °C. The lower organic phase (300 µL) was removed, and the aqueous phase was extracted a second time. The organic phase coming from the second extraction was joined to the first one, dried, and reconstituted in 100 µL of H_2_O:Methanol:Isopropanol:Acetonitrile (7:2:0.5:0.5 *v/v*). For ceramides quantification by LC-MS/MS analysis, the reconstituted sample was diluted 1:4 before injection.

The LC-MS/MS system was a HPLC Alliance HT 2795 Separations Module coupled to Quattro UltimaPt (Waters Corporation, Milford, MA, USA), operating in positive electrospray ionization mode with MassLynxv4.1 software (Waters, Milford, MA, USA). For HPLC analysis, the Ascentis Express Fused-CoreC18 2.7 µm, 7.5 cm × 2.1 mm column with C18 4 × 2.0 mm as precolumn (Phenomenex^®^, Torrance, CA, USA) was used. Twenty µL was injected using water (solvent A) and Methanol:Isopropanol:Acetonitrile 4:1:1 *v/v* (solvent B), both with 0.1% formic acid as the mobile phase. The initial solvent composition was 50% A and 50% B, and the mobile phase gradient profile involved the following steps: increasing to100% B after 4 min, holding for 14 min before returning to the initial gradient. The total run time was 25 min. The flow rate was 0.25 mL/min, and the column was maintained at 30 °C. The parameters for the MRM experiments created for each analyte are summarized in Supplementary Table S6. Data processing and quantification were performed using the QuanLynx 4.1 software (Waters Corporation, Milford, MA, USA).

Moreover, serum sphingomyelins were also monitored as previously described [25].

### 4.9. Targeted Metabolomics for AAs and ACCs Determination

The determination of 14 amino acids (AAs), free carnitine (C0), 35 acylcarnitines (ACCs), succinyl acetone (SA), 2 nucleosides and 4 phospholipids was performed in serum samples (7 µL) by DIMS analysis, using the NeoBase 2 Non-derivatized MSMS Kit (Perkin Elmer Life and Analytical Sciences, Turku, Finland), as already reported [26,27]. Direct infusion mass spectrometry (DIMS) analysis was carried out for the determination of the metabolite profiles in serum samples by an Ultra Performance Liquid Chromatography Tandem Quadrupole Mass Spectrometry UPLC-MS-MS system (Acquity UPLC I-Class coupled to a Xevo TQD, Waters Corp., Manchester, UK). The instrument operated in positive electrospray ionization, with MRM as acquisition mode, using MassLynx V4.2 Software (Waters Corp.) with automatic data processing by NeoLynx (Waters Corp.). Autosampler injections of 10 μL were made into the ion source directly by a narrow peek tube. The total run time was 1.1 min, injection to injection. The capillary voltage was set to 3.50 kV, the source temperature was 120 °C, the desolvation temperature was 350 °C, and the collision cell gas pressure was 3.0 × 10^−3^ mbar Argon. The list of analyzed metabolites and a detailed description of the abbreviations used in the text are available in Supplementary Table S7. In particular, sample preparation has been already reported [28,29].

### 4.10. Statistics

The acquired data of steroids, estrogens, and lipids were processed using MarkerLynx software (Waters, Milford, MA, USA), whereas AAs and ACCs were quantified by NeoLynx software (Waters, Milford, MA, USA). Partial least squares Discriminant Analysis (PLS-DA) was performed using SIMCA-P + 11.0 software (Umetrics AB, Umea, Sweden). The D’Agostino and Pearson omnibus normality test and the Kruskal-Wallis test were used for comparisons between all times of pregnancy and the post-partum period, while Dunns’ test was applied as post-hoc test, considering that only six samples were collected each time from the same MuS women. The histograms related to steroids, estrogens, and lipid levels, as well as Pearson correlation between EDSS and the metabolites levels, were obtained using GraphPad Prism (GraphPad software, Inc., La Jolla, CA, USA). Cluster analysis of AAs and ACCs, visualized as heatmap, was carried out by using the “time series data and two-factor analysis” tool of Metaboanalyst 3.0 software. Rank correlations between metabolites and progesterone levels were performed by a Spearman non-parametric correlation test, using MedCalc 7.6 (MedCalc software, Demo Version, 8400 Ostend, Belgium). A 95% of confidence interval was assumed for each test. Values of *p* < 0.05 were considered significant.

## Figures and Tables

**Table 1:** Details of the most important metabolic values in PLS-DA classification. The *p*-Values of each time comparison are reported in the table, as well as not significant (ns) *p*-values. T1 vs. T2: First Trimester vs. Second Trimester; T2 vs. T3: Second Trimester vs. Third Trimester; T1 vs. T3: First Trimester vs. Third Trimester; T1 vs. post-partum (P-P): First Trimester vs. post-partum; T2 vs. P-P: Second Trimester vs. P-P; T3 vs. P-P: Third Trimester vs. P-P; the data were obtained by unpaired *t* test with Welch’s correction. VIP: Variable Importance of Projection; Sa: Sphinganine; Sa1P: Sphinganine-1-Phosphate; C16Cer: Ceramide (d18:1/16:0); C24dHCer: Dihydroceramide (d18:1/24:1): C0: Free Carnitine; So1P: Sphingosine-1-Phosphate; C16GlcCer: Glucosylceramide (d18:1/16:0); C24: Tetracosanoylcarnitine; C2: Acetylcarnitine; C18Cer: Ceramide (d18:1/18:0); C18:1: Octadecenoylcarnitine.

Metabolite	VIP Value	Kruskal–Wallis *p*-Value	T1 vs. T2	T2 vs. T3	T1 vs. T3	T1 vs. P-P	T2 vs. P-P	T3 vs. P-P
**So**	2.29	<0.0001	0.0001	ns	0.05	0.05	ns	ns
**Sa1P**	1.80	<0.0001	0.0001	0.01	ns	0.01	ns	ns
**C16Cer**	1.60	<0.01	0.05	ns	0.05	ns	ns	ns
**C24dHCer**	1.57	<0.001	0.01	ns	0.01	0.05	ns	ns
**C0**	1.41	<0.0001	ns	ns	ns	0.05	0.0001	0.0001
**So1P**	1.41	<0.01	0.0001	ns	ns	ns	0.05	ns
**C16GlcCer**	1.32	<0.0001	ns	ns	0.05	ns	0.01	0.0001
**C24**	1.32	<0.001	ns	ns	0.01	0.01	ns	ns
**C2**	1.32	<0.0001	ns	ns	ns	0.01	0.0001	0.0001
**GLY**	1.27	<0.001	ns	ns	ns	0.01	0.0001	ns
**ALA**	1.25	<0.01	ns	ns	0.05	0.01	ns	ns
**C18Cer**	1.23	<0.01	ns	ns	0.01	ns	ns	ns
**GLNLYS**	1.21	<0.001	ns	ns	ns	0.05	0.0001	0.05
**TYR**	1.09	<0.001	ns	ns	ns	0.05	0.01	0.05
**PRO**	1.08	<0.01	ns	ns	ns	0.01	0.05	ns
**Sa**	1.02	<0.0001	0.01	0.01	ns	0.0001	ns	0.0001
**C18:1**	1.00	<0.01	ns	ns	ns	ns	0.01	ns

**Table 2:** Observed differences, in terms of *p*-value as well as not significant (ns) *p*-value, in each time comparison for steroids and estrogens quantified by Liquid Chromatography Tandem Mass Spectrometry (LC-MS/MS) analyses. First Trimester versus Second Trimester: T1 vs. T2; Second Trimester versus Third Trimester: T2 vs. T3; First Trimester versus Third Trimester: T1 vs. T3; First Trimester versus Post-Partum: T1 vs. P-P; Second Trimester versus Post-Partum: T2 vs. P-P; Third Trimester versus Post-Partum: T3 vs. P-P; Data were obtained by the Kruskal-Wallis test and Dunns’ post hoc test. CORT: Cortisol; CCONE: Corticosterone; 11-DECOL: 11-Deoxycortysol; ADIONE: 4-Androstene-3,17-dione; TESTO: Testosterone; 17-OHP: 17α-hydroxyprogesterone; PROG: Progesterone; E2: Estradiol; E1: Estrone.

Metabolite	ANOVA	T1 vs. T2	T2 vs. T3	T1 vs. T3	T1 vs. P-P	T2 vs. P-P	T3 vs. P-P
**CORT**	**<0.0001**	<0.01	ns	<0.05	ns	<0.0001	<0.0001
**CCONE**	**<0.001**	ns	ns	<0.05	ns	<0.05	<0.01
**11-DECOL**	**<0.01**	ns	ns	ns	ns	<0.01	<0.05
**ADIONE**	**<0.005**	ns	ns	ns	<0.05	<0.01	ns
**TESTO**	**<0.0001**	ns	ns	ns	<0.01	<0.0001	<0.01
**17-OHP**	**<0.0001**	ns	ns	ns	<0.05	<0.0001	<0.0001
**PROG**	**<0.0001**	ns	ns	<0.01	ns	<0.0001	<0.0001
**E2**	**<0.0001**	ns	ns	<0.01	ns	<0.0001	<0.0001
**E1**	**<0.0001**	ns	ns	ns	ns	<0.0001	<0.0001

## Supplementary Materials

Supplementary materials can be found at http://www.mdpi.com/1422-0067/19/11/3589/s1.
