# Supplementary material for: Metabolomic Signature in Sera of Multiple Sclerosis Patients during Pregnancy
Source: Int J Mol Sci. 2018 Nov 14;19(11):3589. doi: 10.3390/ijms19113589 (PMC6274842; doi:10.3390/ijms19113589)
Supplement: Supplementary file 1 [file ijms-19-03589-s001.pdf]

Metabolomic signature in sera of Multiple Sclerosis patients during pregnancy

Claudia Rossi[1, 2,] , Ilaria Cicalini[1,2], Mirco Zucchelli[1,2], Maria di Ioia[3], Marco Onofrj[3], Luca Federici[1, 2], Piero Del Boccio[2,4] and Damiana Pieragostino[1, 2, *]

[1]Department of Medical, Oral and Biotechnological Sciences, "G. d'Annunzio" University of Chieti-Pescara, Chieti, Italy.[2]Analytical Biochemistry and Proteomics Laboratory, Research Centre on Aging and Translational Medicine (Ce.S.I-MeT), University "G. d'Annunzio" of Chieti-Pescara, Chieti, Italy[3]Department of Neurosciences and Imaging, University "G. d'Annunzio" of Chieti-Pescara, Chieti, Italy

[4]Department of Pharmacy, University ''G. d'Annunzio'' of Chieti-Pescara, Chieti, Italy

*Corresponding Author:

Damiana Pieragostino,
PhD
Analytical Biochemistry and Proteomics Laboratory,
Research Centre on Aging and Translational Medicine (Ce.S.I-MeT)
"G. d'Annunzio" University of Chieti,Via Luigi Polacchi 66100 Chieti, ItalyE-mail:
dpieragostino@unich.itPhone: +39 0871 541596Fax: +39 0871 5411

Table S1: Table reports Rank correlation between PROG and all the metabolites quantified in the study using GraphPad Prism, by a Spearman non-parametric correlation test. The P-value and the related R2 are reported in the Table for each correlation.Significant P-value are in bold.   * Indicates the metabolites which showed the best correlations with PROG.

	**P-value**	**R2**
**SM(d18:0/22:2OH))**	***0.000233872***	**-0.5565466**
**SM(d18:1/22:1)**	***0.000419227***	**-0.5313177**
**SM(d18:0/22:0) ***	***0.00118983***	**-0.5000256**
**Sa ***	***0.01065382***	**0.3900909**
So	0.1030722	0.2550533
So1P	0.1385614	0.2324206
Sa1P	0.7329037	-0.05425701
**C16Cer ***	***0.02537462***	**0.3447216**
C16dHCer	0.2012269	0.2012581
**C18Cer**	***0.03145823***	**0.3324681**
**C16GlcCer ***	***3.73325E-07***	**0.6926081**
C22Cer	0.3074177	-0.1613245
C24Cer	0.1546681	-0.2235659
C24dHCer	0.1951887	0.2039278
ALA	0.4303731	-0.1249697
**ARG**	***0.04165123***	**-0.3157514**
**CIT**	***0.009835111***	**-0.3939792**
**GLN\LYS**	***0.002104789***	**-0.4613329**
GLU	0.09199832	-0.263329
**GLY**	***0.00164687***	**-0.4709086**
**LEU\ILE\PRO-OH**	***0.02541142***	**-0.3446405**
**MET**	***0.04071935***	**-0.3171309**
**ORN**	***0.000776151***	**-0.4986616**
PHE	0.687072	-0.06402668
**PRO**	***0.01759879***	**-0.3646032**
**TYR ***	***1.51946E-05***	**-0.6141368**
**VAL**	***0.002495868***	**-0.4545164**
SA	0.9170961	-0.01656047
**C0 ***	***2.01988E-09***	**-0.7727834**
**C2**	***5.54504E-07***	**-0.6852512**
**C3**	***4.26248E-05***	**-0.5877723**
**C4**	***0.002386757***	**-0.4563178**
C4DC\C5OH	0.07914212	-0.273966
**C5**	***0.003590937***	**-0.4394861**
C5:1	0.3344819	-0.1526537
C5DC\C6OH	0.8729983	0.02542902
**C6DC**	***0.01063388***	**-0.3901825**
C8	0.2438052	-0.1838553
**C8:1**	***0.02126855***	**-0.35446**
C10	0.3384958	-0.1514056
C10:1	0.5499362	-0.09490666
C10:2	0.161919	-0.2197994
C12	0.4372343	-0.1231327
C12:1	0.7072321	-0.05970265
C14	0.07657495	-0.2762495
C14:1	0.2298096	-0.1893272
C14:2	0.3231024	0.1562437
**C16**	***0.007240189***	**-0.4084737**
C16:1	0.1217909	-0.2424924
**C18**	***0.01826321***	**-0.3626425**
**C18:1**	***0.007500257***	**-0.4068351**
**C18:2**	***0.01083267***	**-0.3892756**

C24	0.6924239	0.06287444
C26	0.3936955	-0.1350826
**C20:0-LPC**	***0.000324744***	**-0.5281254**
**C22:0-LPC**	***0.00902675***	**-0.3981006**

Table S2:

**Samples Code**	**Pregnancy**	**Age**	**EDSS during pregnancy**	**Post-Partum Relapses**	**Post-Partum EDDS**
SM01	First	31	1	no	1
SM02	Twins	33	nd	nd	nd
SM03	First	30	nd	nd	nd
SM04	First	32	2	yes	2
SM05	First	32	1.0	yes	1
SM06	Second	32	4	yes	6
SM07	First	26	1	no	1
SM08	Second	34	2	no	2.5
SM09	First	37	2	yes	3.5
SM10	First	30	1	no	1
SM11	First	37	1.5	no	1.5
SM12	First	26	3.5	no	2.5

Table S2: Clinical Details of enrolled patients: nd :Not determined ; EDSS (Expanded Disability Status Scale)

Table S3

Analyte	Calibration Levels (µM)							QC Levels (µM)		
	L1	L2	L3	L4	L5	L6	L7	QC1	QC2	QC3
CORT	0.004	0.01	0.023	0.05	0.14	0.35	0.88	0.012	0.06	0.44
CCONE	0.0008	0.002	0.005	0.011	0.03	0.072	0.18	0.002	0.01	0.09
11-DECOL	0.0002	0.0005	0.0015	0.003	0.008	0.020	0.052	0.0007	0.003	0.025
ADIONE	0.0002	0.0006	0.0017	0.004	0.01	0.025	0.063	0.0008	0.004	0.03
TESTO	0.0001	0.0002	0.0007	0.001	0.004	0.010	0.025	0.00035	0.001	0.01
17-OHP	0.0003	0.0008	0.002	0.005	0.013	0.032	0.078	0.001	0.005	0.04
PROG	0.0003	0.0009	0.002	0.005	0.013	0.033	0.084	0.001	0.005	0.04

Table S3: Concentration levels (µM) for calibrators and QC materials of each steroid monitored in the LC-MS/MS method of analysis are summarized

Table S4:

Analyte	Calibration Levels (µM)							QC Levels (µM)		
	L1	L2	L3	L4	L5	L6	L7	QC1	QC2	QC3
Sa	0.0003	0.0016	0.003	0.016	0.03	0.08	0.16	0.002	0.03	0.14
So	0.0006	0.016	0.003	0.016	0.03	0.08	0.16	0.002	0.03	0.14
So1P	0.0026	0.026	0.13	0.26	0.40	0.65	0.92	0.1	0.33	0.78
Sa1P	0.0026	0.026	0.13	0.26	0.40	0.65	0.92	0.1	0.33	0.78
C16Cer	0.02	0.093	0.18	0.28	0.37	0.46	0.55	0.14	0.31	0.47
C16dHCer	0.02	0.092	0.18	0.28	0.37	0.46	0.55	0.14	0.35	0.47
C18Cer	0.02	0.088	0.18	0.26	0.35	0.44	0.53	0.13	0.30	0.45
C16GlcCer	0.0015	0.014	0.07	0.14	0.21	0.28	0.35	0.05	0.18	0.42
C22Cer	0.08	0.16	0.32	0.80	0.96	1.28	1.60	0.24	0.82	1.36
C24Cer	0.08	0.15	0.30	0.80	0.92	1.23	1.5	0.23	0.78	1.30
C24dHCer	0.015	0.07	0.15	0.23	0.30	0.38	0.5	0.11	0.26	0.39

Table S4: Concentration levels (µM) for calibrators and QC materials of each sphingolipids and ceramides monitored in the LC-MS/MS method of analysis are summarized.

Table S5:

MRM Function	Analyte	Transitions (*m*/*z*)	Cone Volts	Coll Energy (eV)
	E2	506.2 > 171.1	110	32
	$^{2}H_{4}$-E2	510.2 > 171.1	110	32
	E1	504.2 > 171.1	110	32
	$^{2}H_{4}$-E1	508.2 > 171.1	110	32

Table S5. MS/MS operating conditions. Multiple reaction monitoring(MRM) functions and settings for detection of estrogens are shown.

Table S6:

MRM Function	Time window (min)	Analyte	Transitions (m/z)	Cone Volts	Coll Energy (eV)
1	3-7	Sa	302.3 > 284.2	14	50
		d 17:0 Sa	288.2 >270.2	14	
2	3-7	So	300.2 > 282.2	10	50
		d 17:1 So	286.2 > 268.2	11	
3	4-8,5	Sa1P	382.2 > 284.2	13	50
		d 17:0 Sa1P	368.2 > 270.2	13	
4	4,5-10	So1P	380.2 > 264.2	15	50
		d 17:1 So1P	366.2 > 250.2	15	
5	7-13,5	C16Cer	538.4 > 264.2	19	50
		C17Cer	552.5 > 264.2	18	
		C18Cer	566.5 > 264.2	21	
		C16dHCer	540.5 > 522.5	18	
		C16GlcCer	700.5 > 520.4	15	
		C17GlcCer	714.6 > 264.2	18	
6	12-20	C22Cer	622.5 > 264.2	23	50
		C24Cer	650.6 > 264.2	22	
		C24dHCer	652.5 > 634.7	23	

Table S6. MS/MS operating conditions. Multiple reaction monitoring(MRM) functions and settings for detection of sphingolipids and ceramides are shown.

Table S7:

Abbreviation Amino acids and Internal Standards	Amino acids	Transition	Cone potential	Collision energy
Ala<br>**$^{2}H_{3}$-Ala**	Alanine	90.1>44.0<br>93.1>47.1	22	8
Arg<br>**$^{2}H_{4}$, ^{13}C-Arg**	Arginine	175.1>70.1<br>180.1>75.1	34	21
Asa<br>**$^{2}H_{4}$, ^{13}C-Arg**	Argininosuccinic acid	291.1>70.1<br>180.1>75.1	42<br>34	30<br>21
Cit<br>**2H2-Cit**	Citrulline	176.1>113.1<br>178.1>115.1	24	16
Gly<br>**^{15}N,2-^{13}C-Gly**	Glycine	76.0>30.0<br>78.0>32.0	22	7
Leu/Ile/Pro-OH<br>**$^{2}H_{3}$-Leu**	Leucine/Isoleucine/Hydroxyproline	132.1>86.1<br>135.1>89.1	24	10
Met<br>**$^{2}H_{3}$-Met**	Methionine	150.1>104.1<br>153.1>107.1	24	10
Orn<br>**$^{2}H_{6}$-Orn**	Ornithine	133.1>70.1<br>139.1>76.1	22	16
Phe<br>**$^{13}C_{6}$-Phe**	Phenylalanine	166.1>120.1<br>172.1>126.1	25	12
Tyr<br>**$^{13}C_{6}$-Tyr**	Tyrosine	182.1>136.1<br>188.1>142.1	26	12
Val<br>**^{15}N,2-$^{13}C_{5}$-Val**	Valine	118.1>72.1<br>124.1>77.1	23	10
**Pro**<br>**$^{13}C_{5}$-Pro**	Proline	116.1>70.1<br>121.1>74.1	28	12
**Gln/Lys**<br>**$^{13}C_{5}$-Gln**	Glutamine/Lysine	147.1>84.0<br>152.1>88.1	22	16
**Glu**<br>**$^{13}C_{5}$-Gln**	Glutamic acid	148.1>84.0<br>152.1>88.1	24<br>22	14<br>16
**Abbreviation Acylcarnitines and Internal Standards**	**Acylcarnitines**	**Transition**	**Cone potential**	**Collision energy**
C0<br>**$^{2}H_{9}$-C0**	Free Carnitine	162.1>1030<br>171.2>103.0	38	16
C2<br>**$^{2}H_{3}$-C0**	Acetylcarnitine	204.1>85.0<br>207.1>85.0	34	18
C3<br>**$^{2}H_{3}$-C3**	Propionylcarnitine	218.1>85.0<br>221.2>85.0	32	18
C4 C3DC/C4OH<br>**$^{2}H_{3}$-C4**	ButyrylcarnitineMalonylcarnitine/3-Hydroxy-butyrylcarnitine	232.2>85.0<br>248.1>85.0<br>235.2>85.0	36	18
C5 C5:1 C4DC/C5OH<br>**$^{2}H_{9}$-C5**	ValerylcarnitineTiglylcarnitineMethylmalonylcarnitine/3-Hydroxy-valerylcarnitine	246.2>85.0<br>244.2>85.0<br>262.1>85.0<br>255.2>85.0	38	20
C6<br>**$^{2}H_{3}$-C6**	Hexanoylcarnitine	260.2>85.0<br>263.2>85.0	37	20
C5DC/C6OH<br>C6DC<br>**$^{2}H_{6}$-C5DC**	Glutarylcarnitine/3-Hydroxy-hexanoylcarnitine<br>Adipylcarnitine	276.2>85.0<br>290.2>85.0<br>282.2>85.0	40	24
C8:1 C8<br>**$^{2}H_{3}$-C8**	Octenoylcarnitine<br>Octanoylcarnitine	286.2>85.0<br>288.2>85.0<br>291.2>85.0	42	22

C10<br>C10:2<br>C10:1 **$^{2}H_{3}$-C10**	Decanoylcarnitine<br>Decadienoylcarnitine<br>Decenoylcarnitine	316.2>85.0<br>312.2>85.0<br>314.2>85.0<br>319.3>85.0	45	22
C12<br>C12:1<br>**$^{2}H_{3}$-C12**	Dodecenoylcarnitine<br>Dodecanoylcarnitine	344.3>85.0<br>342.3>85.0<br>347.3>85.0	46	24
C14C14:1<br>C14:2<br>C14OH<br>**$^{2}H_{3}$-C14**	Tetradecanoylcarnitine (myristoylcarnitine)<br>Tetradecenoylcarnitine<br>Tetradecadienoylcarnitine<br>3-Hydroxy-tetradecanoylcarnitine	372.3>85.0<br>370.3>85.0<br>368.3>85.0<br>388.3>85.0<br>375.3>85.0	52	25
C16:1<br>C16<br>C16OH<br>**$^{2}H_{3}$-C16**	Hexadecenoylcarnitine<br>Hexadecanoylcarnitine (Palmitoylcarnitine)<br>3-Hydroxy-hexadecanoylcarnitine	398.3>85.0<br>400.3>85.0<br>416.3>85.0<br>403.4>85.0	55	26
C16:1OH/C17<br>**$^{2}H_{3}$-C16**	3-Hydroxy-hexadecenoylcarnitine	414.3>85.0<br>403.4>85.0	55	26
C18<br>C18:1<br>C18:2<br>C18:1OH<br>C18OH<br>C18:2OH<br>**$^{2}H_{3}$-C18**	Octadecanoylcarnitine (Stearoylcarnitine)<br>Octadecenoylcarnitine (Oleylcarnitine)<br>Octadecadienoylcarnitine (Linoleylcarnitine)<br>3-Hydroxy-octadecenoylcarnitine<br>3-Hydroxy-octadecanoylcarnitine3-Hydroxy-octadecadienoylcarnitine	428.4>85.0<br>426.4>85.0<br>424.3>85.0<br>442.4>85.0<br>444.4>85.0<br>440.3>85.0<br>431.4>85.2	56	28
C20<br>**$^{2}H_{3}$-C26**	Eicosanoylcarnitine (Arachidoylcarnitine)	456.4>85.0<br>543.5>85.0	69	34
C22<br>**$^{2}H_{3}$-C26**	Docosanoylcarnitine (Behenoylcarnitine)	484.4>85.0<br>543.5>85.0	69	34
C24<br>**$^{2}H_{3}$-C26**	Tetracosanoylcarnitine (Lignoceroylcarnitine)	512.5>85.0<br>543.5>85.0	69	34
C26<br>**$^{2}H_{3}$-C26**	Hexacosanoylcarnitine (Cerotoylcarnitine)	540.5>85.0<br>543.5>85.0	69	34
**Abbreviation Ketones and Internal Standards**	**Ketones**	**Transition**	**Cone potential**	**Collision energy**
SA<br>**$^{13}C_{5}$-MPP^{2}**	Succinylacetone	155.1>109.1<br>160.1>114.1	24	22
**Abbreviation Nucleosides and Internal Standards**	**Nucleosides**	**Transition**	**Cone potential**	**Collision energy**
ADO<br>**$^{13}C_{5}$-ADO**	Adenosine	268.1>136.1<br>273.1>136.1	32	18
D-ADO<br>**$^{13}C_{5}$-dADO**	2'-deoxyadenosine	252.1>136.1<br>257.1>136.1	29	14
**AbbreviationLysophospholipidsand Internal Standards**	**Lysophospholipids**	**Transition**	**Cone potential**	**Collision energy**
C20:0-LPC<br>**$^{2}H_{4}$-C26:0-LPC**	C20:0 lysophosphatidylcholine	552.4>104.1<br>640.5>104.1	74	30
C22:0-LPC<br>**$^{2}H_{4}$-C26:0-LPC**	C22:0 lysophosphatidylcholine	580.4>104.1<br>640.5>104.1	74	30
C24:0-LPC<br>**$^{2}H_{4}$-C26:0-LPC**	C24:0 lysophosphatidylcholine	608.5>104.1<br>640.5>104.1	74	30

C26:0-LPC<br>**$^{2}H_{4}$-C26:0-LPC**	C26:0 lysophosphatidylcholine	636.5>104.1<br>640.5>104.1	74	30

Table S7. Electrospray ionization mass spectrometry (ESI-MS) acquisition parameters employed for the analysis of whole serum amino acids (AAs), free carnitine (C0),acylcarnitines (ACCs), ketones as succinylacetone (SA), nucleosides and lysophospholipids. MS/MS transitions for each analysed metabolites and the corresponding internal standard (IS, shown in bold), the optimal cone potential (V), and collision energy (eV) are shown for each analyte. The capillary potential was 3.5 kV.

Figure S1:

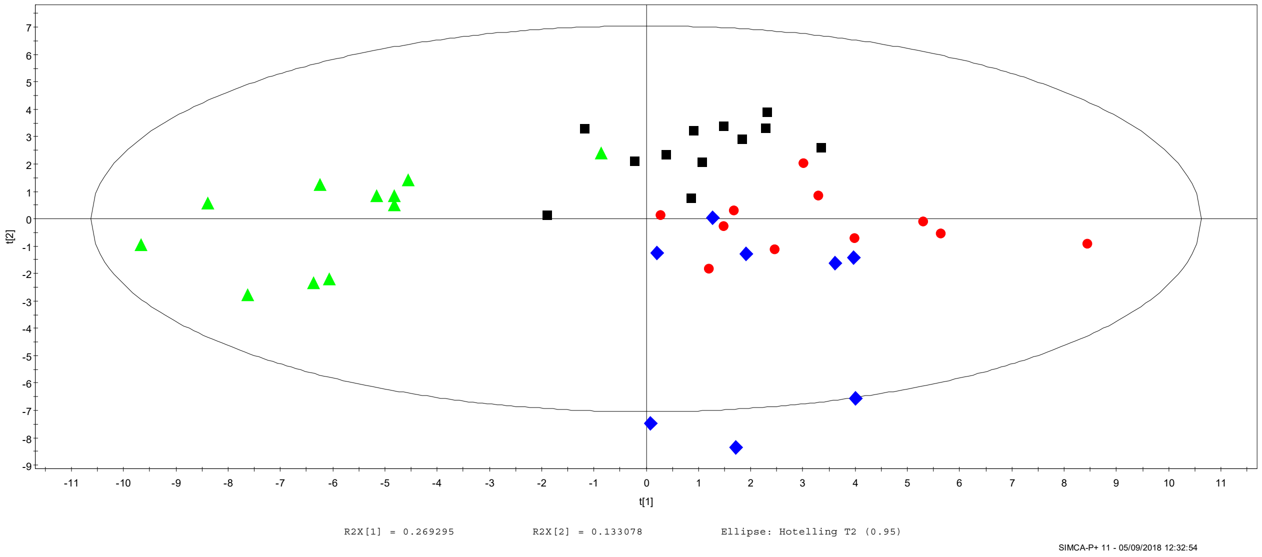

Serum metabolomics investigation by PLS-DA

Figure S1: shows the Scores scatter plot calculated on two components by using 42 observations of 12 MuS cases divided into 4 different times of pregnancy groups. The classification is based on each metabolite concentration. The Partial Last Square Discriminant Analysis (PLS-DA) are used to classify serum from MuS patients in each time group as follow: black boxes (first trimester of pregnancy); red dots (second trimester of pregnancy); blue diamonds (third trimester of pregnancy); green triangle (post-partum) obtained by R2Y=0.448 and Q2(cum)=0.345.

Figure S2

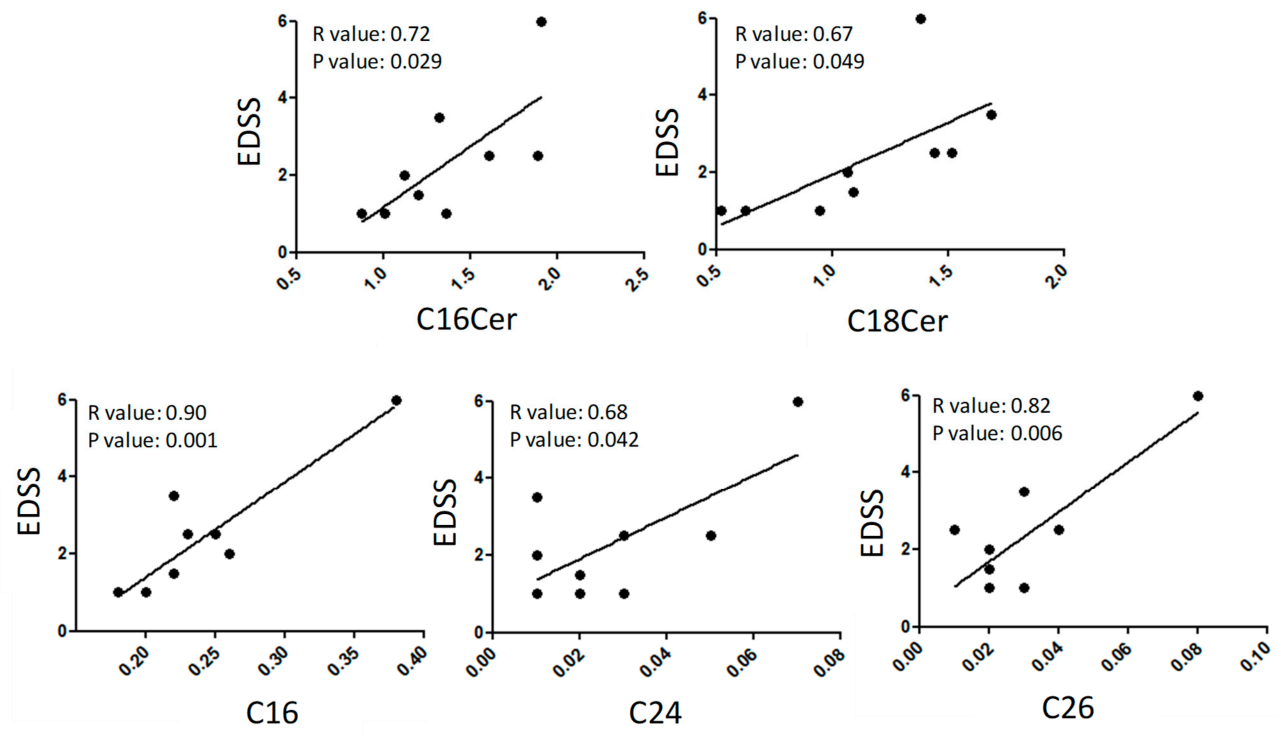

Correlation analysis between EDSS and Metabolic Profile

Figure S2: We performed Pearson correlation between EDSS and Metabolites levels in the post-partum. Correlation graphs were reported in Figure S1, showing strong correlation between EDSS and C16Cer C18Cer,C16 ,C24 and C26 .
